# Pontoon trap for salmon and trout equipped with a seal exclusion device catches larger salmons

**DOI:** 10.1371/journal.pone.0201164

**Published:** 2018-07-26

**Authors:** Linda Calamnius, Mikael Lundin, Arne Fjälling, Sara Königson

**Affiliations:** 1 Biology Department, Faculty of Health and Occupational Studies, University of Gävle, Gävle, Sweden; 2 Institute of Freshwater Research, Department of Aquatic Resources, Swedish University of Agricultural Sciences, Stångholmsvägen 2, Stockholm, Sweden; 3 Harmångers Machine & Marine, Stocka, Sweden; 4 Department of Wildlife, Fish and Environmental studies, Swedish University of Agricultural Sciences, Skogsmarksgränd 9, Umeå, Sweden; 5 Institute of Coastal Research, Department of Aquatic Resources, Swedish University of Agricultural Sciences, Lysekil, Sweden; Universita degli Studi di Bari Aldo Moro, ITALY

## Abstract

The growing seal populations of the Baltic have led to more frequent interactions with coastal fisheries. The motivation for seals to interact with fishing gear is high. It provides high densities of fish. A successful means of mitigating the conflict is the pontoon trap. Seal visits here have been frequent. Seals have access to most parts of the trap system including the middle chamber, which is an overhead environment. Concerns have been raised about seals possible entanglement in this specific part of the trap. As a means of keeping seals from entering the middle chamber, two different Seal Exclusion Devices (SEDs) were tested. A diamond mesh SED and a square mesh SED, which was rotated 45°. The aim was to compare the functionality of the different SEDs with respect to seal deterrent abilities and catch composition. The hypothesis tested were (i) that seals would not be able to enter the middle chamber, (ii) that the catch would increase and (iii) that the SED would deter larger fish from swimming into the middle chamber. Catch data and underwater film were collected. Larger salmons were caught in traps equipped with SEDs. The SEDs did not affect the number of caught fish or the total catch per soak day.

## Introduction

In the middle of the 1970’s the Baltic seal populations were considered to be critically endangered due to a combination of excessive culling and environmental toxins [1–3]. Due to a successful collaborative management of the countries surrounding the Baltic, the seal populations of the Baltic have since recovered. The present annual rate of increase of the three seal populations is estimated to be 8% for the grey seal (*Halichoerus grypus*), 4.5% for the ringed seal (*Pusa hispida*) and 9% for the harbour seal (*Phoca vitulina)* [4]. The population growth of the seals have however led to an increase of seals interacting with fishing gear in the coastal fisheries [5,6]. In the Baltic it is predominantly the grey seal which interacts with the fishing gear [7,8]. The motivation for seals to visit fishing gear is high. Fishing gear such as trap-nets provide easy access to high densities of prey [9].

Pinnipeds interacting with fishing gear is a global problem and occurs e.g. in Tasmania [10], Chile [11], the North American East and West coast [9,12], Scotland [13] and in the Baltic Sea [14]. They cause damage to gear and catch, generate less evident catch losses [15] and occasionally become entangled in the gear, succumbing to accidental death [16–18]. As a means to minimize bycatch (non-target species caught in fishing gear) and to mitigate the interactions between pinnipeds and fisheries or fish farms, a variety of mitigation means have been tried and implemented with varying degrees of success.

The mitigation methods can be divided into two major groups; lethal and non-lethal. The two lethal methods are culling of specific animals [19]) and hunting as population control. This can also be called large scale culling where random animals are killed [20]. There is a wide variety of non-lethal methods. They are composed of deterring methods, physical barriers or removal and relocation of the animals. Deterring methods are e.g. the emittance of sharp sounds using an Acoustic Harassment Device (AHD, [21]) or an Acoustic Deterrent Device (ADD, [22]), lights [23], electrical gradient [24], emetics (taste aversion, [25,26]), visual deterrent in the form of killer whale decoys [11], tactile harassment (e. g. cattle prods, bean bag loads) or hazing (vessel chasing, [27]).

It is a formidable task to deter seals from fishing gear, due to possible habituating or conditioning effects [28]. It has been suggested that using physical barriers might be advantageous in remote areas, where human activities are infrequent [29]. Physical barriers includes predator netting, set traps and Seal Exclusion Devices (SEDs). A predator net is an extra net barrier distancing pinnipeds from the net cage in e.g. fish farms [30]. SEDs are installed in existing fishing gear and functions as both an escape means to reduce bycatch in trawls [17,18,31,32] or to exclude seals from the catch in cod pots, while concurrently reducing bycatch [16].

A successful means of non-lethal mitigation used in the Baltic is the pontoon trap [6,33–35]. This type of set trap is a stationary and passive fishing gear. It includes a leader net and a system of progressively smaller chambers, making it easy for the fish to enter, but not to exit. It was originally developed to replace traditional set traps for salmon (*Salmo salar*) and brown trout (*Salmo trutta*). In recent years, models have been developed for vendace (*Coregonus albula*), for herring (*Clupea harengus*) and for other fish species. It is included in the category of Low Impact Fuel Efficient (LIFE) fishing capture techniques [36].

The pontoon trap has been considered to be a cause for concern in bycatch issues [37]. Vanhatalo et al., (2014, [37]) estimated the annual bycatch of grey seals in set nets in Swedish, Finnish and Estonian waters to be 2 280 animals which represents around 88% of the total bycatch of seals.

**Fig 1:**
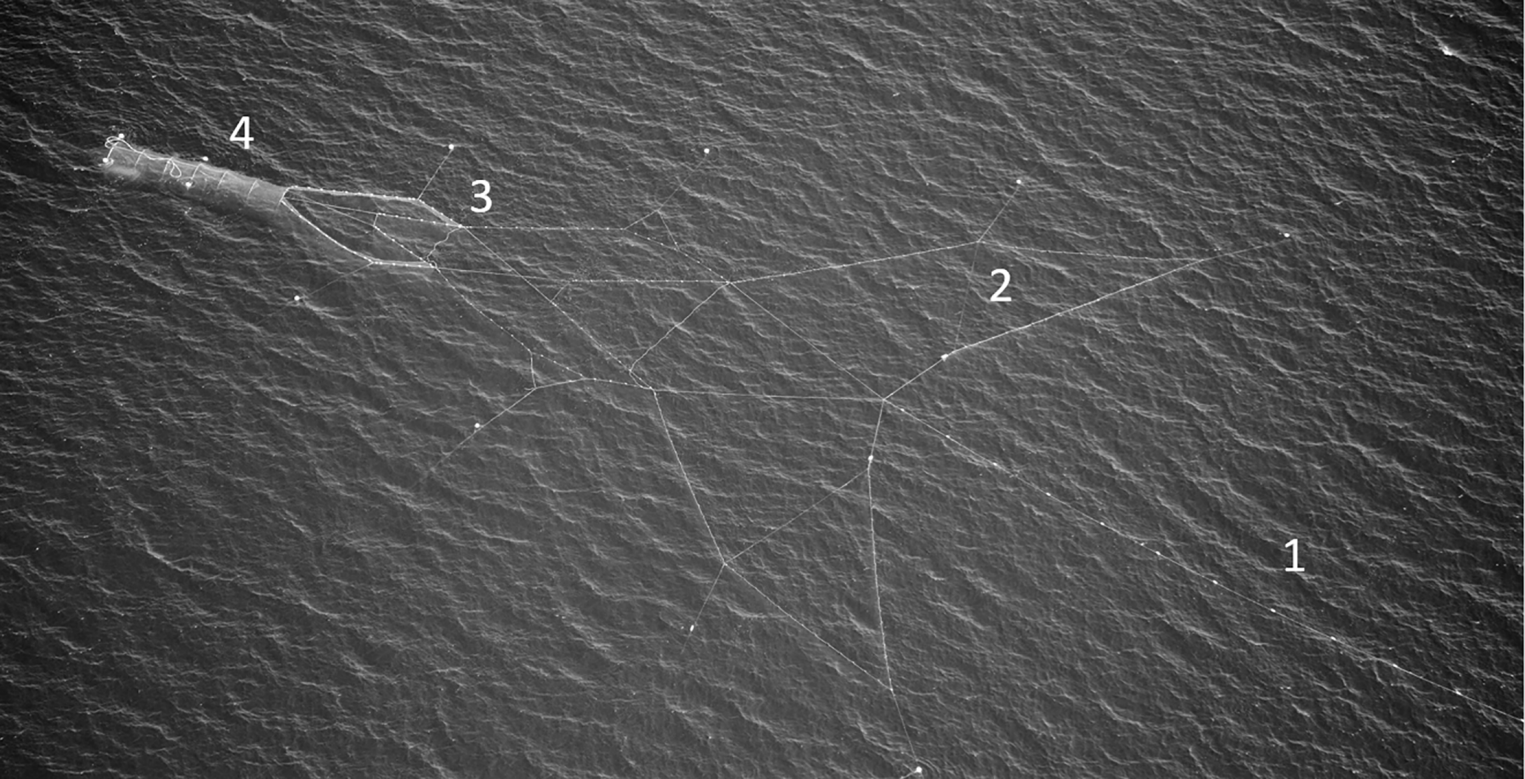
Aerial view of complete pontoon trap for salmon and brown trout. (1) Leader net, (2) wings, (3) first and second adapter and (4) middle and fish chamber, which is an overhead environment. The total length of the middle and fish chamber is 12.5 m (*Photo*: *Swedish Coastguard*).

Even if the accumulated catch in the fish chamber of the pontoon trap is protected, seals have access to the preceding middle chamber, which has a net roof and no direct access to the surface (Fig 1). Seal visits to these parts of the trap are frequent [7]. An obvious method to lessen the interaction of seals in the pontoon trap (bycatch and seal depredation) is to prevent their access into the middle chamber.

Since the introduction of the pontoon traps in the late 1990’s [33], there has been further developments in its design, to prevent seals from accessing fish. Seals have used the traps to their advantage, chasing fish into the side panels (wings) of the trap [6]. By using larger mesh in the side panels (400 mm instead of the earlier 200 mm), it allowed chased fish to pass through the mesh, thus escaping the pursuant seal [6]. Another modification has been to use double netting in the fish bag (i.e. fish chamber), instead of the traditional single walled netting [29]. Fish caught in traps with double walled netting suffered less seal induced damage (1–2%, compared to 30–50%, [29]). The same study also installed a type of SED (a frame with a grid). Catches were greater in traps with this grid [29].

Designing a SED is a compromise between not having any adverse effect on the catch, while simultaneously preventing seals from entering [16,34,35]. The size and shape of the openings in the SED are critical. Smaller openings may lead to a better retention of fish [16]. If the openings are too small, or the wires or twine in the entrance too conspicuous, the fish might turn and swim out. The shape of the openings should be as inconspicuous as possible to minimize its visual impression on the fish. SED in cod pots have achieved desired results of good catchability while preventing seals from entering [16]. Prevention of seals access to fish traps will reduce seal-induced damage and stress on caught fish [38] and bycatch of seals [16]. In this paper, we describe and evaluate two different designs of SEDs in pontoon traps for salmon and brown trout by looking at the individual size of the caught fish, the total catch and the amount of seal interactions.

## Materials and methods

**Fig 2:**
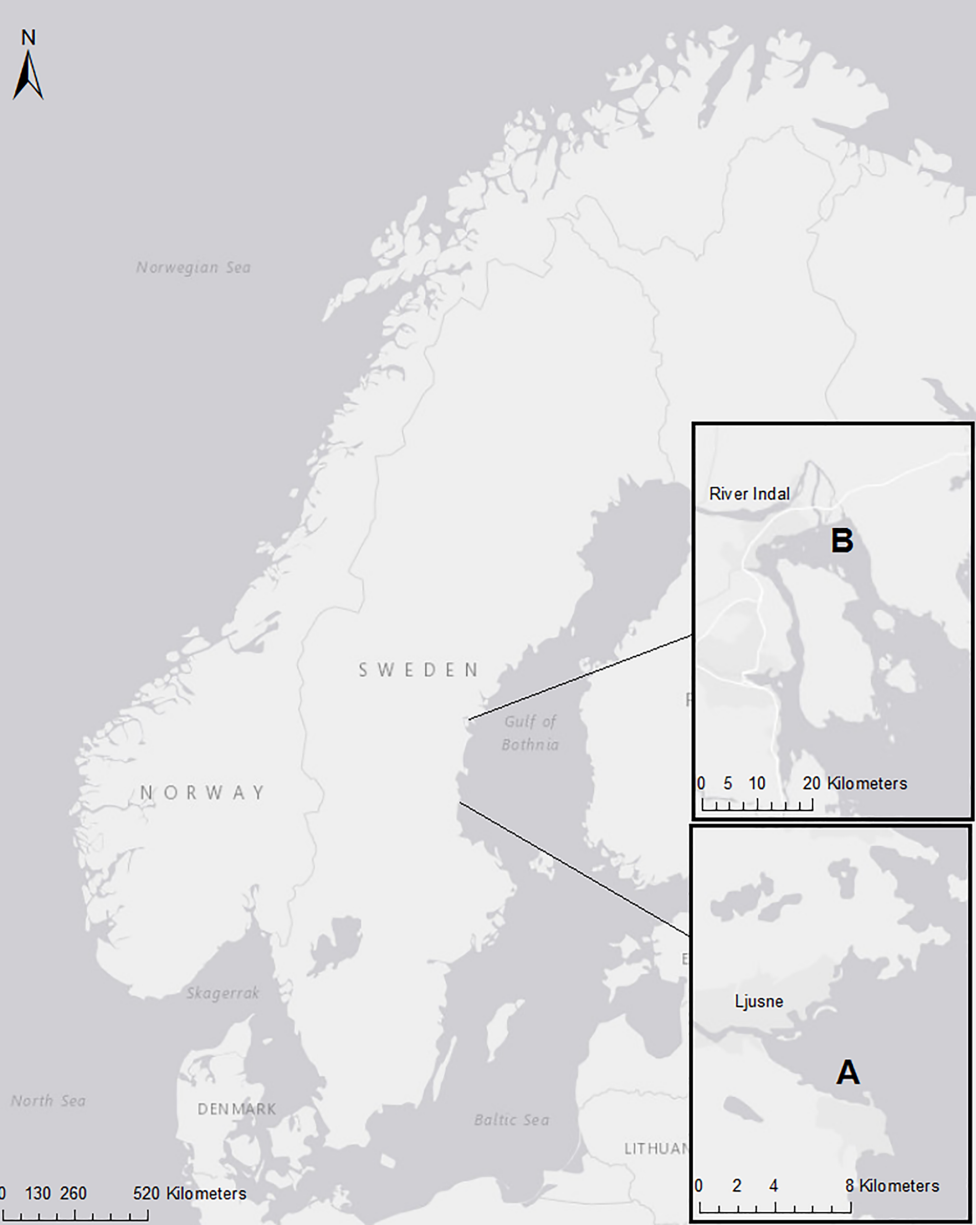
Location of the traps. (A) At Ljusne and (B) at River Indal. The distance between Ljusne and River Indal is approximately 145 km. Map by ArcGIS software Desktop 10.5.1. 7333 [39].

The study was conducted in Sweden in two different locations (Fig 2). The type of fishing gear used were pontoon traps for salmon and trout (Fig 1). The final part of the trap, the fish chamber is lifted to the surface by inflating the pontoons under it and the catch is harvested from a chute with a hatch. A detailed description of the pontoon trap can be found in Lunneryd et al. (2003, [6]), Suuronen et al., (2006, [29]) or Hemmingsson et al., (2008, [33]).

### Ethical permit

The study was undertaken in collaboration with commercial fishers. Observational studies on wild animals in Sweden are allowed without permit, under the condition that the animals are not caught, not killed, not herded and not exposed to any kind of treatment which could lead to suffering (Regulations and general advice on laboratory animals of 2015, [40]). The seals in this study were not caught, killed, herded or exposed to any type of treatment that could have led to suffering. One of the purposes of using a SED is to reduce bycatch of seals. The fish in the study were part of the catch in a commercial fishery and an ethical permit was therefore not required. All efforts to minimize the suffering of the fish were still applied.

### Fishing permit

The study was conducted on private property. Permissions to fish were granted from the respective owners or leasees of the waters, who also participated in the study (S1 Table). The commercial salmon fishery in Sweden is regulated by the Swedish Agency for Marine and Water Management. When the quota for salmon was reached, the fishery was stopped.

### Set up of experiment

The traps used were pontoon traps for salmon and trout and were set on their migratory routes. Two traps were used in both locations. The traps altered status as experimental or control trap, as the setups (diamond or square mesh SEDs) were installed or removed in the entrance to the middle chamber. The control trap was an empty frame, i.e. an open entrance. The interval between the switches was one week. The fish traps were randomly assigned, control or experimental.

At Ljusne, the distance between the traps was 800 m. The length of the leader nets were 100 m and 140 m. The study started on the 26^th^ of June and ended on the 25^th^ of July (duration 29 days). Switching SEDs or control in the traps occurred three times.

At River Indal, the distance between the traps was 250 m. The leader nets were 130 m long for both traps. The study was started on the 26^th^ of June and was aborted on the 11^th^ of July (duration 15 days). Two exchanges of SEDs or control took place during this period, with one trap being a control trap during the first two weeks.

### Seal exclusion devices

**Fig 3:**
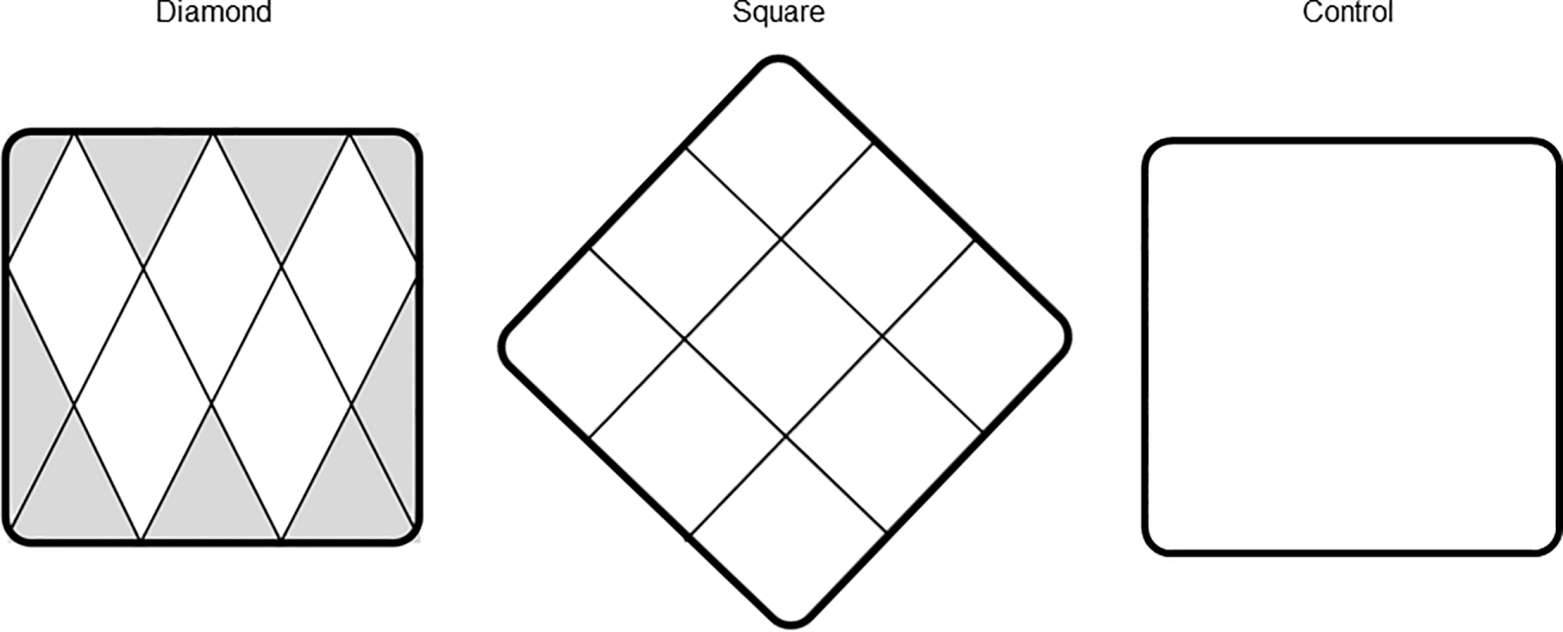
The two Seal Exclusion Devices (SEDs) and the control frame. The diamond mesh SED, the square mesh SED and the control. The control was an open aluminium frame. The grey area in the diamond mesh SED indicates half- or quarter mesh.

**Fig 4:**
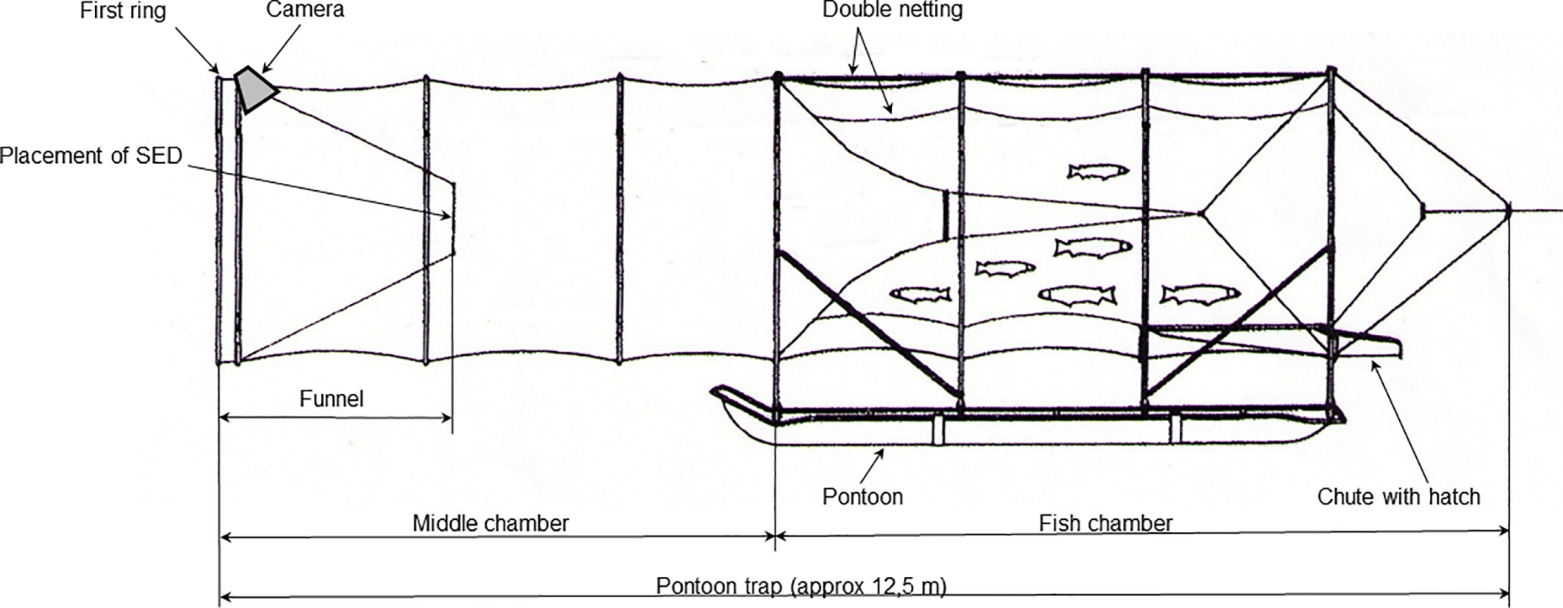
Side view of middle and fish chamber, with camera placement. This part of the trap corresponds to position 4, in Fig 1.

Two SEDs were used. A diamond mesh SED and a square mesh SED (Fig 3). The control was an open frame. The SEDs and the control consisted of a frame, 800 x 800 mm, made from 12 mm aluminium pipe. The mesh in the SEDs was 3 mm green, twisted Dyneema^®^. This is a strong synthetic fibre with characteristics similar to that of Kevlar^®^. Both arrangements of the SEDs constitute a potential hinder for fish as a visually induced aversion or a physical obstruction. The SEDs were mounted in the entrance to the middle chamber (Fig 4).

Stretched mesh in the diamond mesh SED was 550 mm. The diagonal height was 500 mm, the width was 270 mm and the circumference was 1140 mm. The frame with the square mesh SED was rotated 45°. Stretched mesh was 534 mm. The diagonal mesh height and width was 377 mm, with a circumference of 1067 mm. Stretched mesh is an internationally used measuring method. It measures the distance between the centre of the two knots when fully stretched [41].

### Data collection, camera system and image analysis

Data was collected per species with individual weight of each fish and number of fish per harvest. Video recordings were made with three camera systems. Batteries and recorders were placed in waterproof boxes, which were placed in a dinghy attached to the trap. An underwater Sony Super 1/3” HAD 700 TVL CCD video camera was used, with 50 m cable and an SSD recording unit. It was powered by a 12 V, 50 Ah LiFePO4 battery. The camera was placed in one of the traps. It was attached to the first ring and aimed at the entrance. The film speed was set at two frames per second. Each frame was provided with a time stamp. Windows Media Player was used for viewing the films.

The recordings were screened manually and sequences with seal visits were tagged with date and time. A seal visit was defined as when part of the seal’s body extended into the funnel. The collected data was organized in a Windows Excel spreadsheet.

### Statistical analyses

The software used for the statistical analyses was IBM SPSS, version 24.0.0.2 for Windows.

The catch data was calculated separately per species. The differences between the SEDs and the control were analysed with respect to the weight of the individual fish, number of caught fish, Catch Per Unit Effort (CPUE) and Weight Per Unit Effort (WPUE). CPUE and WPUE were respectively defined as the total numbers of fish or the total weight of the catch per soak day in one trap.

A Kruskal-Wallis test was applied to the catch data. A Chi-square test was applied to the data of seal visits.

## Results

The traps in Ljusne were harvested on 15 occasions each. Both traps in each pair were harvested on the same day.

The study at River Indal was aborted due to one or more seals that damaged both catch and gear in the control trap. The damage was extensive. The seal(s) had entered the middle chamber, proceeded into the fish chamber and destroyed the funnel. Both the diamond mesh SED and the square mesh SEDs were destroyed approximately two weeks after the study began. The resulting hole in the diamond mesh SED was large enough to allow a seal to enter. It is not possible to determine whether the seal(s) did or did not proceed into the middle chamber. These experiments took place in a subsistence fishery and it was deemed too detrimental to the fishery to continue with the experiment at this location. The participating fisher did not want to use a trap without a SED.

### Individual weight of salmon and trout, CPUE and WPUE

**Fig 5:**
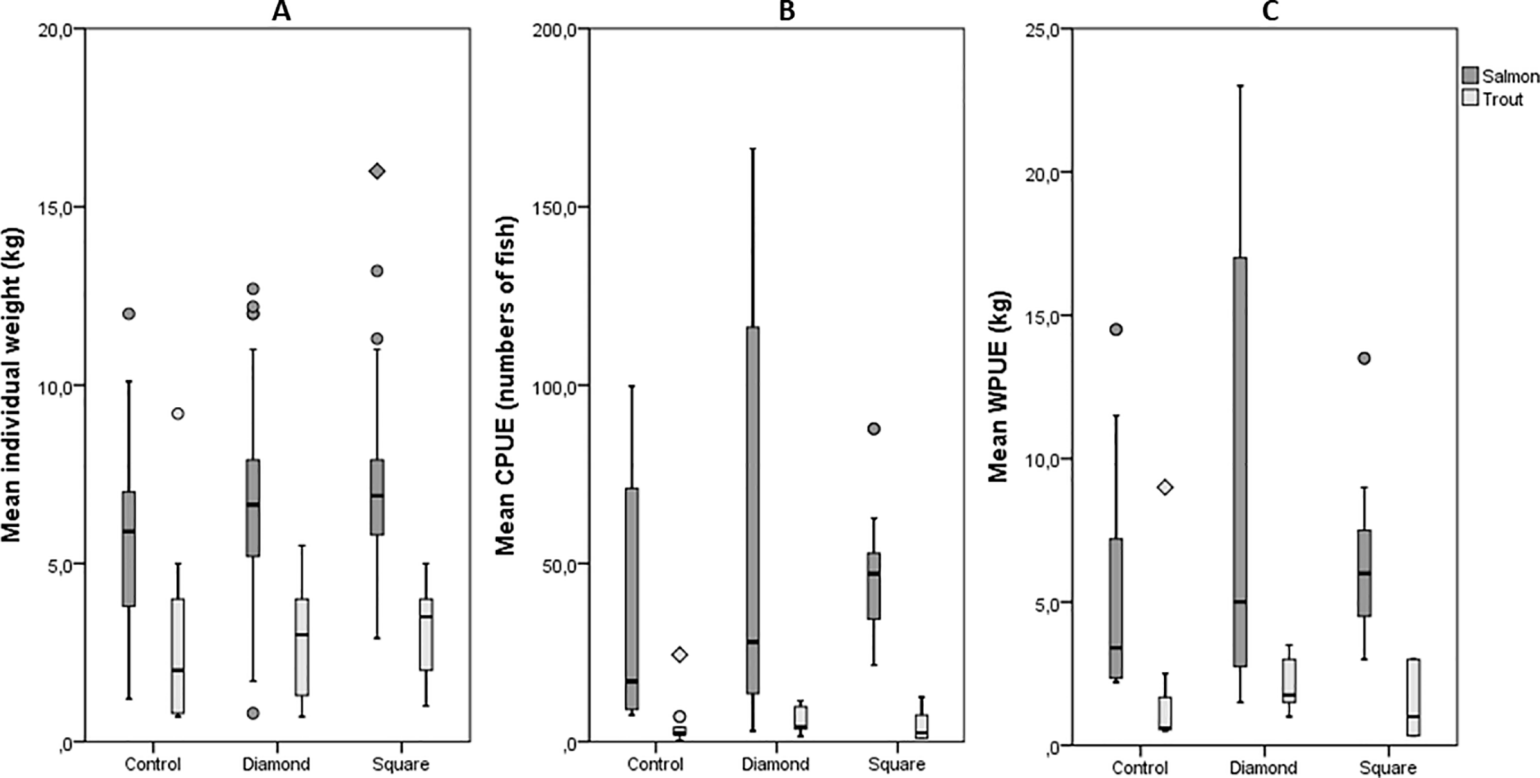
**Box plots of (A) the individual weight of the fish, (B) the CPUE and (C) the WPUE between the different treatments.** The box contains 50% of all values. The black line within the box represents the median. The whiskers above and below indicates the 10th and 90th percentiles respectively. Each outlier is represented either by a circle (1.5 times the interquartile range) or by a rotated square (3 times the interquartile range).

Heavier salmons were caught in traps with a SED than in the control trap, *X*^*2*^(2) = 21.927, *p* < .001 (Fig 5). The average weight of trout between the groups was not significantly different, *X*^*2*^(2) = 2.011, *p* = .366.

There was no significant difference in CPUE between the SEDs and the control trap for either salmon (*X*^*2*^(2) = 5.156, *p* = .076) or for trout (*X*^*2*^(2) = 5.537, *p* = .063). There was no significant difference in WPUE between the SEDs and the control trap for either salmon (*X*^*2*^(2) = 2.917, *p* = .233) or for trout (*X*^*2*^(2) = 3.868, *p* = .145). Catch data is included in supporting information (S2 Table).

### Seal visits

There was one filmed seal visit throughout the experiment (Table 1). It was at the square mesh SED. The seal stayed at the SED for a little over 5 minutes. During this period it tore apart the twine in the SED. There were no filmed seal visits in the trap with the diamond mesh SED or in the control trap. In all, seals destroyed three of the four SEDs used during the experiment. Two were with the square mesh (one each in River Indal and Ljusne) and one with the diamond mesh (River Indal).

**Table 1:** Comparison of frequency of seal visits between experimental and control traps.

Test	Seal visits (no)	Amount of film (hr)	Total time in trap or by SED (mm:ss)	Proportion of time by SED per filmed hour (%)
Square mesh SED	1	134	05:13	6.5 x 10^−2^
Diamond mesh SED	0	91	00:00	0
Control	0	91	00:00	0

A Chi-square test was performed on the number of filmed hours in which a seal had been present in the funnel. There were no significant differences in visits between traps equipped with a SED or the control trap, df = 2, *X*^*2*^ = 1.44, *p* = 0.49.

There was no bycatch of seals in the middle chamber in any of the traps. Unfortunately, two male seals were bycaught in earlier parts of the trap. One of the seals had become entangled in the leader net near the entrance to one of the wings. The other was entangled in the first adapter. Both seals had an estimated weight of slightly over 100 kg each.

## Discussion

### Using a SED matters on the size of the salmons

Using a SED in the entrance of the middle chamber resulted in heavier salmons. It is likely that there was a retaining effect [16,34] by the SEDs on the larger salmons. The fish were not hindered from entering, but were inhibited from exiting. In a previous study by Lehtonen and Suuronen (2004 [34]), the weight of the salmon catch was almost twice as much in the trap equipped with a grid (i.e. SED), than in the control trap. The grid in their study was manufactured with 2 mm steel wires. The current study used a 3 mm twine. A thicker twine makes a greater visual impression. This was counterbalanced by the use of the diamond mesh or square mesh, which makes less visual impression than a grid. Diamond or square mesh has wider open areas, when compared to a grid. The greater catch in Lehtonen and Suuronen (2004, [34]) suggests that the SED in the form of a grid, was more efficient at retaining fish.

The use of SEDs did not have any effect on the individual weight of the trout. In an earlier pilot project, using the diamond mesh SED, heavier trout were caught in the control trap. The low proportion of larger trout in the trap with the diamond mesh SED, suggested that it had a deterring effect on larger trout. In a SED with diamond mesh, some mesh will be half- or quarter mesh (Fig 3). The total area of full mesh in the diamond mesh SED was 56%. Half- and quarter-mesh make a more visual impression than full mesh and might be perceived as an obstacle by hesitant fish. It is imperative to make use of the full area of the entrance. In a square frame, with square mesh, the full area of the opening will be used, with no resulting half- or quarter mesh.

The SEDs did not affect either the CPUE or the WPUE for either of the species, suggesting that the presence of a SED did not have any adverse effects on the total catch.

### Did the SED prevent presence of seals in the traps?

There was only one seal visit throughout the entire study. It was therefore not possible to deduct whether the SED had any deterrent effect on seals or not. Our expectation was that the number of visits in the control trap would be in parity with an earlier study by Königson et al., (2013 [7]), where no SEDs were used. The location for this study was approximately 30 km south of the study area at River Indal. In Königson et al., (2013, [7]) there were 83, 89 and 428 and visits in three salmon traps. It is not possible to draw any conclusions regarding the differences between the earlier study and the current one. The crew of visiting seals has in all probability changed between then and now.

A parameter which should be kept in mind is that grey seals can become 35–40 years old [42]. Their longevity has implications for designing fishing gear. It is specialised individuals which visits fishing gear and they constitute less than 1% of the seal population [43]. These visiting seals will over time gain experience of how the catch can be reached, e.g. by tearing through the SEDs as was done in this study. Their behaviour is adaptable when faced with preventive measures [9] and they are difficult to exclude from fishing gear [44]. As a predator it is imperative to be inventive and at the forefront of finding new methods of reaching the prey. Wild grey seals have well documented learning abilities and quickly adapt their foraging forays, in parallel with the advent of new mitigation measures. They have learned to open a hatch in a fish trap [45], to connect sounds from acoustic deterrent devices, acoustic fish tags or boats with foraging opportunities [46,47], to associate buoys with fishing gear [48] and to associate gillnets or fish farms with food [22,49].

The seals learning ability and adaptability is one of the major challenges when designing new mitigation means or modifying existing fishing gear. If one part of a fish trap is made inaccessible they will shift their attention to other parts of the trap [34,35], or possibly destroy gear as occurred in the current study. In the study by Lehtonen and Suuronen [29] seals were observed exploring the grid without causing damage. Seal induced damage occurred then on the netting of the adapters or on the fish bag (i.e. fish chamber). In the present study, seal induced damage was not observed on the netting. Instead, the SEDs were subject to damage by seals on several occasions. The torn SEDs attests to the seals high motivation of getting to the catch, suggesting that a change of material in the mesh is necessary.

Applying a SED in the entrance of the middle chamber will prevent most seals from entering an overhead environment, thus reducing the number of seals that are bycaught. The SED does not prevent fish from receiving seal-inflicted injuries prior to entering the final parts. It does prevent seals from further injuring and stressing fish that are caught in the middle chamber of the trap. Presence of seals will affect the catch negatively [15]. Severe stress drains muscular energy and increases the production of lactic acid [50]. A reduction of stress is an important factor in maintaining high-quality flesh in fish [51] as fish exposed to stress have a lower condition factor [52].

## Conclusions

With the results from this study, the recommendation for commercial fishers is that using a SED has a positive effect on the individual size of salmons. They were not hindered by the presence of a SED. The described SED is low-cost, easy to manufacture and easy to use. It can be mounted and de-mounted in a few minutes in a fishing trap when it is on the surface.

A suggestion for future improvements of the SED is to use steel rods for mesh material, with a spot weld where the rods intersect. Given the seals persevering and adaptable nature, it is a risk that preventing them from entering the middle chamber might result in other parts of the trap becoming their focus of attention.

Mitigating the conflict between fisheries and seals is one of the most difficult questions in the coastal fisheries of the Baltic [53]. So far there is no single measure which has been able to provide complete protection from visiting seals [45]. The seals are proactive, while the commercial fisheries are reactive. With the growing population of seals and their learning capabilities it is of utmost importance to conduct further studies on their behaviour, in order to find innovative mitigating methods, to reduce potential bycatch and keeping the seals at bay leaving the catch to the fishers.

## Supporting information

S1 Table Landowners or leasees of waters. (DOCX)

S2 Table Comparison of catches, between traps with square mesh SED, diamond mesh SED and control trap. (DOCX)

## References

[1] Harding KC, Härkönen TJ. Development in the Baltic Grey Seal (*Halichoerus grypus*) and Ringed Seal (*Phoca hispida*) Populations during the 20th Century. Ambio. 1999;28: 619–627. Available: http://www.jstor.org/stable/4314968

[2] Swedish Agency for Marine and Water Management. Sälpopulationernas tillväxt och utbredning samt effekterna av sälskador i fisket. The growth of the seal populations and the effect of seal induced damage in the fisheries [Internet]. Gothenburg; 2014. Available: https://www.havochvatten.se/hav/uppdrag—kontakt/publikationer/publikationer/2015-01-14-salpopulationernas-tillvaxt-och-utbredning-samt-effekterna-av-salskadorna-i-fisket.html

[3] HELCOM. Hazardous substances in the Baltic Sea—An integrated thematic assessment of hazardous substances in the Baltc Sea. Balt Sea Environ Proc No 120B 2010; 116.

[4] Bäcklin B-M, Moraeus C, Strömberg A, Karlsson O, Härkönen T. Sälpopulationer och sälhälsa. Sealpopulations and seal health. Havet 2015/2016: Om miljötillståndet i svenska havsområden The Sea 2015/2016 About the environmental condition in Swedish marine areas. Stockholm; 2016: 116–118.

[5] Bruckmeier K, Höj Larsen C. Swedish coastal fisheries-From conflict mitigation to participatory management. Mar Policy. 2008;32: 201–211. 10.1016/j.marpol.2007.09.005

[6] Lunneryd S-G, Fjälling A, Westerberg H. A large-mesh salmon trap: a way of mitigating seal impact on a coastal fisher. ICES J Mar Sci. 2003;60: 1194–1199. 10.1016/S1054e3139(03)00145-0 A

[7] Königson S, Fjälling A, Berglind M, Lunneryd S-G. Male gray seals specialize in raiding salmon traps. Fish Res. 2013;148: 117–123. 10.1016/j.fishres.2013.07.014

[8] Jounela P, Suuronen P, Millar RB, Koljonen ML. Interactions between grey seal (*Halichoerus grypus*), Atlantic salmon (*Salmo salar*), and harvest controls on the salmon fishery in the Gulf of Bothnia. ICES J Mar Sci. 2006;63: 936–945. 10.1016/j.icesjms.2006.02.005

[9] Nelson M, Gilbert J, Boyle K. The influence of siting and deterrence methods on seal predation at Atlantic salmon (*Salmo salar*) farms in Maine, 2001–2003. Can J Fish Aquat Sci. 2006;63: 1710–1721. 10.1139/F06-067

[10] Hume F, Pemberton D, Gales R, Brothers N, Greenwood M. Trapping and relocating seals from salmonid fish farms in Tasmania, 1990–2000: was it a success? Pap Proc R Soc Tasmania. 2002;136: 1–6. Available: http://eprints.utas.edu.au/13503/%5Cnhttp://eprints.utas.edu.au/13503/1/2002_Hume_Trapping_rst.pdf

[11] Sepúlveda M, Oliva D. Interactions between South American sea lions *Otaria flavescens* (Shaw) and salmon farms in southern Chile. Aquac Res. 2005;36: 1062–1068. 10.1111/j.1365-2109.2005.01320.x

[12] Jamieson GS, Olesiuk PF. Salmon farm—Pinniped Interactions in British Columbia: An Analysis of Predator Control, its Justification and Alternative Approaches. Research Document 2001/142 [Internet]. Nanaimo; 2001 Available: http://www.dfo-mpo.gc.ca/csas/Csas/DocREC/2001/RES2001_142e.pdf

[13] Butler JRA, Middlemas SJ, Graham IM, Harris RN. Perceptions and costs of seal impacts on Atlantic salmon fisheries in the Moray Firth, Scotland: Implications for the adaptive co-management of seal-fishery conflict. Mar Policy. Elsevier; 2011;35: 317–323. 10.1016/j.marpol.2010.10.011

[14] Bruckmeier K, Westerberg H, Varjopuro R. Baltic Seal Reconciliation in Practice. The Seal Conflict and its Mitigation in Sweden and Finland In: Klenke RA, Ring I, Kranz A, Jepsen N, Rauschmayer F, Henle K, editors. Human-Wildlife Conflicts in Europe Fisheries and Fish-eating Vertebrates as a Model Case. Heidelberrg; 2013 pp. 15–48. 10.1007/978-3-540-34789-7

[15] Fjälling A. The estimation of hidden seal-inflicted losses in the Baltic Sea set-trap salmon fisheries. ICES J Mar Sci. 2005;62: 1630–1635. 10.1016/j.icesjms.2005.02.015

[16] Königson S, Lövgren J, Hjelm J, Ovegård M, Ljunghager F, Lunneryd S-G. Seal exclusion devices in cod pots prevent seal bycatch and affect their catchability of cod. Fish Res. Elsevier B.V.; 2015;167: 114–122. 10.1016/j.fishres.2015.01.013

[17] Tilzey R, Goldsworthy S, Cawthorn M, Calvert N, Hamer D, Russel S, et al Seal-fishery interactions in the winter blue grenadier fishery off west Tasmania and the development of fishing practices and Seal Exclusion Devices to mitigate seal bycatch by factory trawlers. Project no 2001/008. [Internet]. Deakins West; 2006 Available: http://frdc.com.au/research/Final_Reports/2001-008-DLD.pdf

[18] Lyle JM, Willcox ST, Hartmann K, Willcox ST, Hartmann K, Jech JM. Underwater observations of seal–fishery interactions and the effectiveness of an exclusion device in reducing bycatch in a mid-water trawl fishery. Can J Fish Aquat Sci. NRC Research Press; 2016;73: 436–444. 10.1139/cjfas-2015-0273

[19] Linnell JDC, Odden J, Smith ME, Aanes R, Swenson JE. Large carnivores that kill livestock- Do problems individuals really exist?.pdf. Wildl Soc Bull. 1999;27: 698–705.

[20] Würsig B, Gailey G. Marine mammals and aquaculture: conflicts and potential resolutions. Responsible Mar Aquac. 2002; 45–59. 10.1079/9780851996042.0045

[21] Mate BR, Brown RF, Greenlaw CF, Harvey JT, Temte J. An Acoustic Harassment Technique to Reduce Seal Predation on Salmon In: Mate BR, Harvey JT, editors. Acoustical Deterrents in Marine Mammal Conflicts with Fisheries. Newport: Oregon State University; 1986 pp. 23–36. Available: http://nsgl.gso.uri.edu/oresu/oresuw86001/oresuw86001_part2.pdf

[22] Gordon J, Northridge S. Potential impacts of Acoustic Deterrent Devices on Scottish Marine Wildlife Scottish Natural Heritage Commissioned Report Edinburgh; 2002.

[23] Yurk H, Trites AW. Experimental Attempts to Reduce Predation by Harbor Seals on Out-Migrating Juvenile Salmonids. Trans Am Fish Soc. 2000;129: 1360–1366. 10.1577/1548-8659(2000)129<1360:EATRPB>2.0.CO;2

[24] Forrest KW, Cave JD, Michielsens CGJ, Haulena M, Smith D V. Evaluation of an Electric Gradient to Deter Seal Predation on Salmon Caught in Gill-Net Test Fisheries. North Am J Fish Manag. 2009;29: 885–894. 10.1577/M08-083.1

[25] Gearin PJ, Pfeifer R, Jeffries SJ, Johnson MA. NWAFC Processed Report 88–30. Results of the 1986–1987 California Sea Lion—Steelhead trout predation control program at the Hiram M. Chittenden locks. Seattle, Washington; 1988.

[26] Stewardson C, Cawthorn MW. Final Report of the Special SESSFEAG Meeting: Reducing Seal Interactions and Mortalities in the South East Trawl Fisheriy. Technologies to reduce seal-fisheries interaction and mortalities. 2003.

[27] Marine and Marine Industries Council. A Seal/Fishery Interaction Management Strategy: Background Report [Internet]. Hobart; 2002. Available: http://dpipwe.tas.gov.au/Documents/Final-Management-Strategy-(FM).pdf

[28] Götz T, Janik VM. Aversiveness of sounds in phocid seals: psycho-physiological factors, learning processes and motivation. J Exp Biol. 2010;213: 1536–1548. 10.1242/jeb.035535 20400639

[29] Suuronen P, Siira A, Kauppinen T, Riikonen R, Lehtonen E, Harjunpää H. Reduction of seal-induced catch and gear damage by modification of trap-net design: Design principles for a seal-safe trap-net. Fish Res. 2006;79: 129–138. 10.1016/j.fishres.2006.02.014

[30] National Seal Strategy Group, Stewardson C. National Assessment of Interactions between Humans and Seals: Fisheries, Aquaculture and Tourism [Internet]. Canberra, Australia; 2007. Available: http://www.agriculture.gov.au/SiteCollectionDocuments/fisheries/environment/bycatch/sealassessment.pdf

[31] Hamer DJ, Goldsworthy SD. Seal-fishery operational interactions: Identifying the environmental and operational aspects of a trawl fishery that contribute to by-catch and mortality of Australian fur seals (*Arctocephalus pusillus doriferus*). Biol Conserv. 2006;130: 517–529. 10.1016/j.biocon.2006.01.014

[32] Hooper J, Clark JM, Charman C, Agnew D. Seal mitigation measures on trawl vessels fishing for krill in CCAMLR subarea 48.3. CCAMLR Sci. 2005;12: 195–205. Available: https://www.researchgate.net/publication/291211930_Seal_mitigation_measures_on_trawl_vessels_fishing_for_krill_in_CCAMLR_subarea_483

[33] Hemmingsson M, Fjälling A, Lunneryd S-G. The pontoon trap: Description and function of a seal-safe trap-net. Fish Res. 2008;93: 357–359. 10.1016/j.fishres.2008.06.013

[34] Lehtonen E, Suuronen P. Mitigation of seal-induced damage in salmon and whitefish trapnet fisheries by modification of the fish bag. ICES J Mar Sci. 2004;61: 1195–1200. 10.1016/j.icesjms.2004.06.012

[35] Varjopuro R, Salmi P. Complexities in keeping seals away from the catch–building ‘seal-proof’ fishing gear. Mast. 2006;5: 61–86. Available: http://www.marecentre.nl/mast/documents/MAST_Vol_5_1_p61-86.pdf

[36] Suuronen P, Chopin F, Glass C, Løkkeborg S, Matsushita Y, Queirolo D, et al Low impact and fuel efficient fishing-Looking beyond the horizon. Fish Res. Elsevier B.V.; 2012;119–120: 135–146. 10.1016/j.fishres.2011.12.009

[37] Vanhatalo J, Vetemaa M, Herrero A, Aho T, Tiilikainen R. By-catch of grey seals (*Halichoerus grypus*) in Baltic fisheries—A Bayesian analysis of interview survey. PLoS One. 2014;9: 1–17. 10.1371/journal.pone.0113836 25423168 PMC4244152

[38] Lundin M, Calamnius L, Hillström L, Lunneryd S-G. Size selection of herring (*Clupea harengus membras*) in a pontoon trap equipped with a rigid grid. Fish Res. 2011;108: 81–87. 10.1016/j.fishres.2010.12.001

[39] ESRI. ArcGIS Desktop. Redlands, CA, USA: Environmental System Research Institute; 2017.

[40] Swedish Board of Agriculture Statutes. Föreskrifter om ändring i Statens jordbruksverks föreskrifter och allmänna råd (SJVFS 2012:26) om försöksdjur. Regulations on Amendments to the Swedish Board of Agriculture’s Regulations and General Advice (SJVFS 2012: 26) on Laboratory Animals [Internet]. Sweden; 2015 p. 78. doi:ISSN 1102-0970

[41] Food and Agriculture Organization of the United Nations. Fisherman’s Workbook Prado J, Dremiere PY, editors. Food and Agriculture Organization of the United Nations. Oxford: Fishing News Books Ltd; 1990 10.1017/CBO9781107415324.004

[42] Reeves RR, Stewart BS, Clapham PJ, Powell JA. National Audubon Society Guide to Marine Mammals of the World New York: Andrew Stewart Publishing Inc; 2002.

[43] Graham IM, Harris RN, Matejusová I, Middlemas SJ. Do “rogue” seals exist? Implications for seal conservation in the UK. Anim Conserv. 2011;14: 587–598. 10.1111/j.1469-1795.2011.00469.x

[44] Westerberg H, Lunneryd S-G, Fjälling A, Wahlberg M. Reconciling fisheries activities with the conservation of seals throughout the development of new fishing gear: A case study from the Baltic fishery-gray seal conflict In: Nielsen J, Dodson JJ, Friedland K, Hamon TR, Musick J, Verspoor E, editors. Reconciling Fisheries with Conservation, Proceedings of the Fourth World Fisheries Congress, Volume I San Fransisco: American Fisheries Society; 2007 pp. 587–697.

[45] Lehtonen E, Suuronen P. Live-capture of grey seals in a modified salmon trap. Fish Res. 2010;102: 214–216. 10.1016/j.fishres.2009.10.007

[46] Königson S, Fjälling A, Lunneryd S-G. Grey seal induced catch losses in the herring gillnet fisheries in the northern Baltic. NAMMCO Sci Publ. 2007;6: 203–213. Available: http://septentrio.uit.no/index.php/NAMMCOSP/article/view/2735

[47] Stansbury AL, Götz T, Deecke VB, Janik VM. Grey seals use anthropogenic signals from acoustic tags to locate fish: evidence from a simulated foraging task. Proc R Soc B. 2015;282: 1–9. 10.1098/rspb.2014.1595 25411449 PMC4262164

[48] Fjälling A, Kleiner J, Beszczyńska M. Evidence that grey seals (*Halichoerus grypus*) use above-water vision to locate baited buoys. NAMMCO Sci Publ. 2007;6: 215–227.

[49] Jokikokko E, Huhmarniemi A. The large-scale stocking of young anadromous whitefish (*Coregonus lavaretus*) and corresponding catches of returning spawners in the River Tornionjoki, northern Baltic Sea. Fish Manag Ecol. 2014;21: 250–258. 10.1111/fme.12068

[50] Poli BM, Parisi G, Scappini F, Zampacavallo G. Fish welfare and quality as affected by pre- slaughter and slaughter management. Aquaculture International, 13: 29–49. Aquac Int. 2005;13: 29–49.

[51] Lowe TE, Ryder JM, Carragher JF, Wells RMG. Flesh Quality in Snapper, *Pagrus auratus*, Affected by Capture Stress. J Food Sci. 1993;58: 770–773. 10.1111/j.1365-2621.1993.tb09355.x

[52] Lundin M, Calamnius L, Lunneryd S-G. Survival of juvenile herring (*Clupea harengas membras*) after passing through a selection grid in a pontoon trap. Fish Res. 2012;127–128: 83–87. 10.1016/j.fishres.2012.05.009

[53] Varjopuro R. Co-existence of seals and fisheries? Adaptation of a coastal fishery for recovery of the Baltic grey seal. Mar Policy. Elsevier; 2011;35: 450–456. 10.1016/j.marpol.2010.10.023

