# Supplementary material for: Pontoon trap for salmon and trout equipped with a seal exclusion device catches larger salmons
Source: PLoS One. 2018 Jul 26;13(7):e0201164. doi: 10.1371/journal.pone.0201164 (PMC6062063; doi:10.1371/journal.pone.0201164)
Supplement: S1 Table — (DOCX) [file pone.0201164.s001.docx]

| **Location** | **Name and adress** |
| --- | --- |
| **Ljusne** | Magnus Johansson, Gästrikevägen 7, 826 39 Söderhamn, Sweden |
| **River Indal** | Lars Bergman, Dammvägen 1,  861 41 Sörberge, Sweden |
|  |  |
