# Supplementary material for: Pontoon trap for salmon and trout equipped with a seal exclusion device catches larger salmons
Source: PLoS One. 2018 Jul 26;13(7):e0201164. doi: 10.1371/journal.pone.0201164 (PMC6062063; doi:10.1371/journal.pone.0201164)
Supplement: S2 Table — (DOCX) [file pone.0201164.s002.docx]

|  | **Salmon** | | | **Brown trout** | | |
| --- | --- | --- | --- | --- | --- | --- |
|  | **Square mesh SED** | **Diamond mesh SED** | **Control** | **Square mesh SED** | **Diamond mesh SED** | **Control** |
| Total weight of catch (kg) Total weight of catch (%) | 875.1  (34.9 %) | 1080.0  (43.0 %) | 555.2  (22.1 %) | 45.5  (24.0 %) | 77.5  (41.0 %) | 66.2  (35.0 %) |
| Catch (no)  Catch (%) | 125  (31.9 %) | 166  (42.3 %) | 101  (25.8 %) | 14  (20.9 %) | 29  (43.3 %) | 24  (35.8 %) |
| Average weight per fish (kg) | 7.0 | 6.5 | 5.5 | 3.2 | 2.7 | 2.8 |
| S. D. average weight per fish (kg) | 1.9 | 2.2 | 2.3 | 1.4 | 1.5 | 2.2 |
| Min – Max (kg) | 2.9 - 16.0 | 0.8 - 12.7 | 1.2 - 12.0 | 1.0 - 5.0 | 0.7 - 5.5 | 0.7 - 9.2 |
| Average CPUE  (numbers of fish) | 46.1 | 56.8 | 37.0 | 4.6 | 5.6 | 4.4 |
| S. D. CPUE  (numbers of fish) | 19.1 | 57.6 | 35.9 | 4.6 | 3.5 | 5.9 |
| Min – Max CPUE  (numbers of fish) | 21.4 - 87.8 | 3.0 - 166.3 | 7.5 - 99.7 | 1.0 - 12.5 | 1.5 - 11.5 | 0.4 - 24.4 |
